# Fabrication of 3D Capillary Vessel Models with Circulatory Connection Ports

**DOI:** 10.3390/mi9030101

**Published:** 2018-02-28

**Authors:** Mahmoud Gallab, Kyohei Tomita, Seiji Omata, Fumihito Arai

**Affiliations:** 1 Department of Micro-Nano Mechanical Science and Engineering, Nagoya University, Furo-cho, Chikusa-ku, Nagoya 464-8601, Japan; tomita@biorobotics.mech.nagoya-u.ac.jp (K.T.); s-omata@mech.nagoya-u.ac.jp (S.O.); arai@mech.nagoya-u.ac.jp (F.A.); 2 Mechanical Design and Production Engineering Department, Faculty of Engineering, Minia University, Minia 61519, Egypt

**Keywords:** bionic models of blood vessels, femtosecond laser, microfluidic devices, photolithography, 3D microchannel fabrication

## Abstract

Bionic microscopic vessel models can contribute to the development of vascular treatment skills and techniques for clinical training. Most microscopic vessel models are limited to two dimensions, but three-dimensional (3D) models are important for surgery, such as on retina microscopic vessels, for the observation of colon microvessels, for measuring the deformability of red blood cell (RBC), and so on. Therefore, bionic 3D blood vessel models are increasingly in demand. For this reason, it is necessary to establish 3D fabrication techniques for microchannels. In this study, we established two fabrication methods for 3D microfluidic devices for the development of microscopic vessel models. First, we employed an exposure method using photolithographic technology. Second, we employed a 3D method using femtosecond laser and mask hybrid exposure (FMEx). Both methods made it possible to fabricate a millimeter-scale 3D structure with a submicrometer resolution and achieve an easy injection of solution. This is because it was possible to fabricate typical microfluidic channels used for model inlet and outlet ports. Furthermore, in the FMEx method, we employed an acid-diffusion effect using a chemically amplified resist to form a circular channel cross-section. The acid-diffusion effect made it realizable to fabricate a smooth surface independent of the laser scanning line width. Thus, we succeeded in establishing two methods for the fabrication of bionic 3D microfluidic devices with microfluidic channels having diameters of 15–16 µm for mimicking capillary vessels.

## 1. Introduction

Recently developed medical technologies including operative procedures, through blood vessels, and medical equipment are quite rapidly evolving and becoming diverse. Therefore, medical doctors need to learn these technologies with only short-term training. Moreover, the development and evaluations of medical equipment, such as endoscopic imaging systems, also need to be conducted in a short time span. However, it is difficult to use the human body for training in new operative procedures or evaluations of new medical devices or to test a hypothesis, like the interaction between microvascular and circulating cells, because of ethical and safety problems. Instead of evaluations using the human body, they are conventionally conducted with animal samples [1]. However, the structure of animal samples is fundamentally different from that of the human body. Therefore, it is difficult to ensure reproducibility of the results of training or evaluation of medical devices using animal samples. One solution to this problem is the use of a surgical simulator [2]. Microvascular simulators create the environments and conditions for surgical procedures with the goal of improving operative skills and patient safety [3], and can also be used for the evaluation of medical equipment, such as endoscopic imaging systems, or testing a hypothesis of interaction between microvascular and circulating cells. Two basic types of surgical simulators exist. One is computer-based virtual reality (VR) simulators [4,5], and the other is simulation models made from artificial materials [6,7,8,9].

Virtual reality simulators reproduce an environment during surgery using computer graphics. Additionally, some virtual reality simulators can reproduce the sense of touch during surgery by using haptic interfaces. However, VR simulators are not sufficient to evaluate new medical devices. This is because the effects of new medical devices on the human body cannot be reproduced completely without the accumulation of large amounts of data. Thus, VR simulators are not suitable for evaluating medical techniques.

A physical simulation model has the advantage that it can be physically touched, enabling the user to learn the essential sensations of touch or texture. Prototype simulators utilize individual samples that are profoundly reproducible because of well-controlled fabrication methods. Blood flow can also be imitated by flowing a fluid in the vessel models by connecting them to external tubes and pumps. Moreover, the selection of fitting materials allows the simulator to be sterilized and facilitates the reproduction of the effects of medical equipment on the human body. Thus, mock-up simulators can be used in actual operating rooms to assess the performance of medical equipment.

Numerous methods, such as stereolithography, ink-jet rapid prototyping, and photolithography, have been proposed for the fabrication of mock-up simulators. Stereolithography, although applicable for the fabrication of molds, has difficulty in creating hollow structures. S. Ikeda et al. [6] proposed a surgical simulator (Endo Vascular Evaluator) with three-dimensional (3D) blood vessel models that are tailor-made using ink-jet rapid prototyping with wax. However, it is very difficult to fabricate microfluidic devices with diameters smaller than 500 μm because of the brittleness of wax.

**Figure 1:**
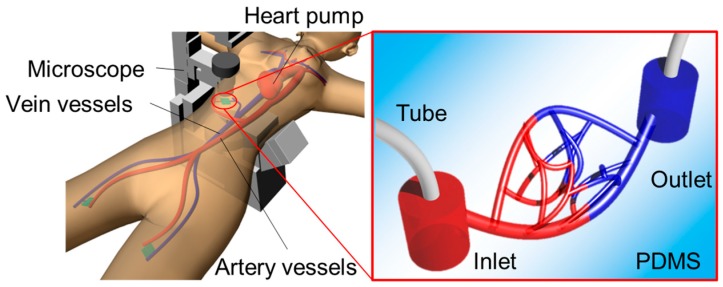
Concept of simulating capillary vessels and arterioles with models.

Recently, 3D printing technologies have begun to make it possible to fabricate a high-resolution microchannel of about 50 μm in diameter [10,11]. However, even smaller microchannels of 10 μm in diameter are needed in order to simulate a bionic capillary vessel environment with realistic diameters and branching structures by applying a micro-nano fabrication technique, as shown in Figure 1 [8]. Photolithography-based fabrication techniques can be applied to fabricate models of smaller vessels. T. Nakano et al. [8] fabricated a microchannel that mimics a fine blood vessel with sizes up to about 10 μm using photolithography techniques and polydimethylsiloxane (PDMS) molding. Although photolithography offers sufficient resolution for the blood vessel model, it can only be applied to two-dimensional (2D) structures. Two-dimensional simulators can be connected to conventional artery models to simulate an arteriole network and blood circulation within it. However, these simulators are limited to two dimensions even though the real vessel is 3D. A capillary vessel simulator, proposed for surgery training, the evaluation of medical equipment, or to test hypotheses concerning microvasculature and its connection with circulating cells, should replicate the 3D structure of an in vivo capillary vessel. In this study, therefore, we propose two fabrication methods for 3D biological simulation models using microchannels to mimic capillary vessels and arterioles.

## 2. Microfluidic Channel Design and Concept

**Figure 2:**
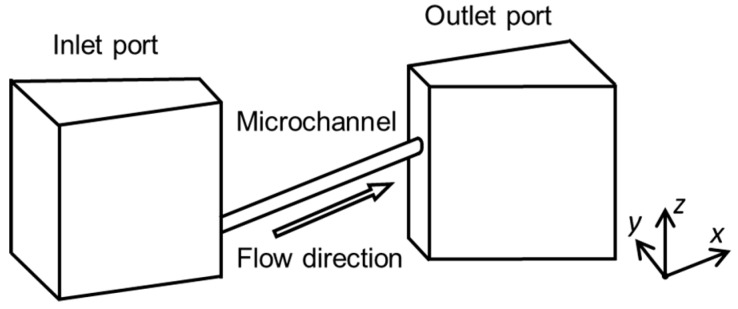
Basic structure of a 3D microchannel.

We designed microchannels for mimicking blood vessels, such as colonic and intraocular retinal microvascular vessels, with two connection ports as the inlet and outlet for the fluid flow. We simplified the shape of the complex actual blood vessel, as shown in Figure 2. We developed two fabrication methods for 3D capillary vessels: (1) photolithography with transfer to a 3D-printed model and (2) femtosecond laser and mask hybrid exposure (FMEx).

### 2.1. Photolithography Method

Photolithography is a fundamental technology for fabricating microchannels, and a high resolution of 1 μm is easily attained [12,13]. Photolithography has been used for fabricating arteriole capillary vessel models [14]. However, this process is not suitable for fabricating 3D models, as it is limited to one flat plane. To achieve a 3D model with photolithography, we propose using photolithography to fabricate a 2D microchannel and then use PDMS (Sylpot 184, Dow Corning Toray Co., Ltd., Tokyo, Japan) molding and the water transfer printing technique to transfer the 2D microfluidic channel to a 3D printed model. First, we fabricate the microchannel on a PDMS sheet by laser lithography and PDMS molding. Next, this thin PDMS sheet is transferred to an angled 3D-printed model, which can be created in any desired shape using 3D printing technology. Third, to make the final surface of the microfluidic device flat, we put the model into a mold, pour PDMS, and remove the excess PDMS by squeegeeing. The result is a 3D microchannel formed at different depths that can be easily controlled. Additionally, the microchannel can be connected to external tubes via the connection ports, as shown in Figure 2, allowing liquid to flow through the microchannel for mimicking blood flow.

### 2.2. Femtosecond Laser Exposure Method

**Figure 3:**
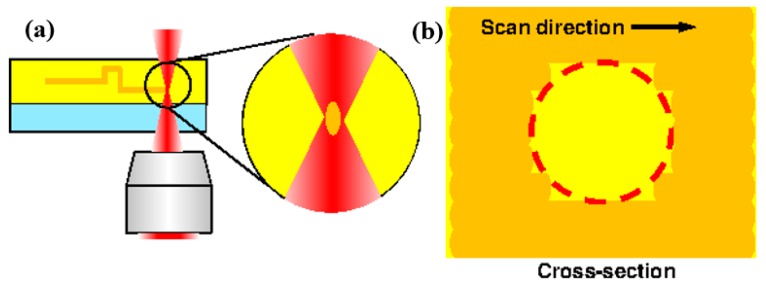
Cause of a rough surface by femtosecond laser exposure. (**a**) Femtosecond laser scanning process; (**b**) Cross-section of the photoresist after scanning.

Here, we propose a method for fabricating a c model with a cross-section close to circular, similar to that of a real blood vessel. During femtosecond laser exposure, the region of the sphere at the focal position of the laser is exposed, as shown in Figure 3. Although it is processed into a 3D shape by repeating laser scanning, surface roughness occurs due to the scan line width and scanning interval. If the minimum line width is reduced by decreasing the laser power or increasing the scanning speed, the surface roughness is also reduced. However, when the scanning interval decreases, the time required for scanning increases. Therefore, we propose to take advantage of the acid diffusion phenomenon by using a chemically amplified resist.

**Figure 4:**
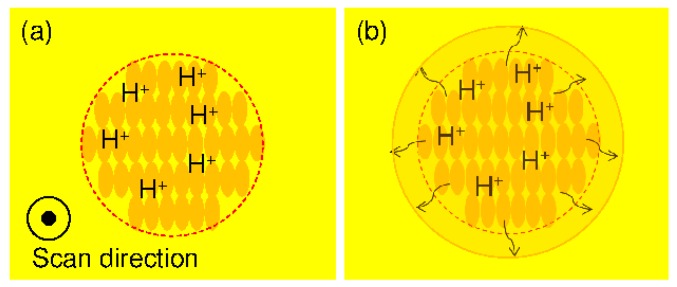
Mechanism for producing a smooth mold surface when using a chemically amplified resist. (**a**) Acid generation after femtosecond laser exposure; (**b**) Acid diffusion during post-exposure bake.

Photoresists are materials that enable fine processing due to changes in the solubility of the region irradiated with light. Chemically amplified resists are a mainstream material for semiconductor fabrication. This material comprises a base polymer, dissolution inhibitor (for positive type resists–negative types use a scrubbing agent), and an acid generator. The acid generated by the exposure is diffused in a post-exposure heating step, that is, the post-exposure bake (PEB), and new acid is generated continuously by an acid catalyst reaction; in the positive type, the exposure dissolves a dissolution inhibitor, and in the negative type, the exposure accelerates a crosslinking reaction to form a pattern. Therefore, compared with a conventional resist, it is possible to form materials with a small exposure dose [15]. This acid-diffusion distance affects resist sensitivity and pattern shape. When the diffusion distance is too long, the resolution decreases because the catalytic reaction of the acid reaches the unexposed region. The diffusion distance of acid is influenced by the residual amount of solvent in the photoresist and the PEB temperature and duration [16,17,18]. Q. Chen et al. [19] effectively used acid-diffusion phenomena and succeeded in producing a smooth shape with the chemically amplified resist SU-8. Here, we propose to make effective use of long-range acid diffusion. The photoresist used is a positive photoresist with high releasability (KMPR, MicroChem Corp., Westborough, MA, USA). After exposing the chemically amplified resist to a femtosecond laser to generate acid (Figure 4a), we actively induce acid long-range diffusion during PEB (Figure 4b). We propose that this concept is used to make a mold of a fine blood vessel with a smooth surface.

## 3. Fabrication methods

### 3.1. Photolithography Exposure Method

To fabricate a microchannel structure on an angled surface, we hydraulically transferred the PDMS pattern, which had the microchannel, to an angled 3D-printed model. Water transfer printing is generally used for printing on curved surfaces [20]. A printed film was floated on water, and the model was pressed onto the surface of the film so that the pattern was uniformly applied to the angled surface. With the water transfer printing process, we realized a fine microchannel with size of ≃15 μm on an angled surface, which is difficult to achieve with conventional fabrication techniques.

**Figure 5:**
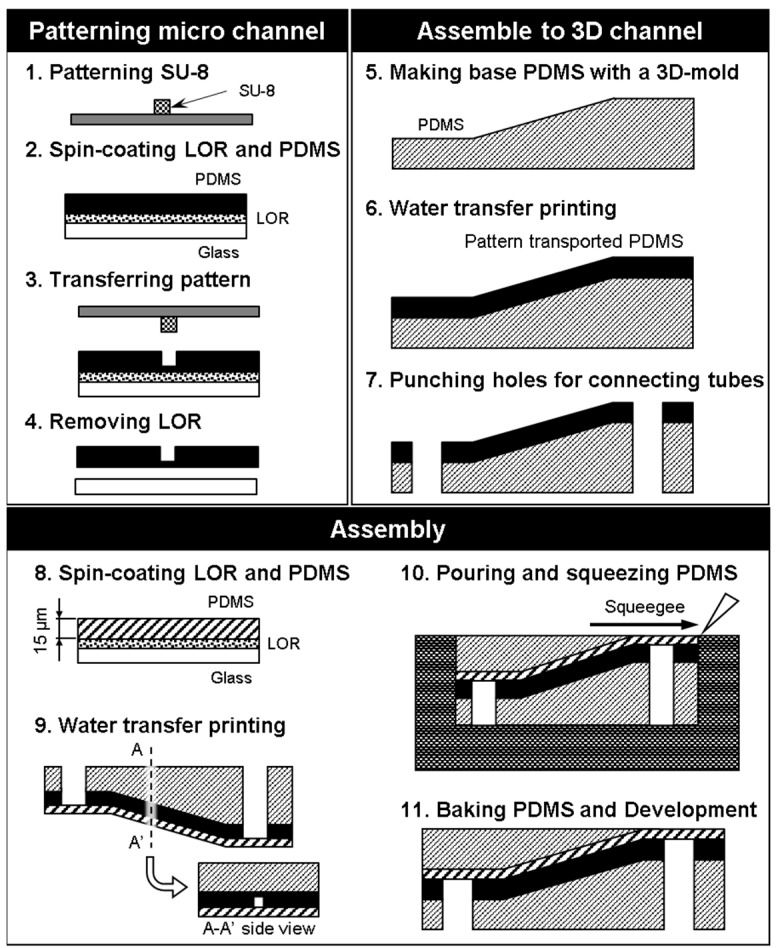
Microvessel model fabrication using photolithography.

The fabrication process steps are summarized below. The step numbers correspond to the numbers in Figure 5.

1. Laser lithography (MA-6, SUSS Micro Tec KK, Kanagawa, Japan) was used to form a pattern in the SU-8 photoresist (Nippon Kayaku Co. Ltd., Tokyo, Japan) on a silicon surface. The exposure time is 9.7 s. The mold was heated using a hot plate at 65 °C for 1 min, and 95 °C for 3 min. Then, the part was developed with propylene glycol monomethyl ether (PM) for 120 s and rinsed with 2-propanol for 60 s. This pattern was used as a mold for the microchannels, and the size of the microchannels could be locally controlled by adjusting the exposure conditions.
2. Spinning coat (2000 rpm for 30 s) was used to apply Lift-off resist (LOR) (Nippon Kayaku Co. Ltd., Tokyo, Japan) and then baked on a hotplate for 10 min at 95 °C. Spin coat PDMS (3000 rpm for 30 s) (Silpot 184, Dow Corning Toray Co. Ltd., Tokyo, Japan) was applied over LOR onto a glass substrate, and then baked on a hotplate for 10 min at 95 °C.
3. The SU-8 mold was pushed onto the spin-coated PDMS and the ensemble was heated to 85 °C for 10 min with a hot plate.
4. The LOR was dissolved with ethanol to free the PDMS sheet from the substrate.
5. A PDMS base was created using a 3D printer (EDEN250, Stratasys Ltd., Eden Prairie, MN, USA).
6. The PDMS sheet and base were treated with O_2_ plasma to activate their surfaces for bonding, and the PDMS sheet was transferred to the PDMS base.
7. Holes were punched into the connection channel to connect external tubes from the bottom side of the model.
8. A thin sheet of PDMS was created by spin-coating to serve as a cover layer for the channel.
9. The thin PDMS sheet was placed over the PDMS sheet containing the microchannel.
10. PDMS was poured and squeezed to form a slab over the angled surface.
11. The assembled PDMS model was baked in the oven for 20 min at 85 °C, and the mold was removed.

In this fabrication process, a 15-μm-wide microchannel could be fabricated by using laser photolithography (step 1 in Figure 5). The patterning on an oblique structure was done using water transfer printing (steps 5 and 6). Furthermore, the thickness of the cover layer was controlled by changing the spin-coating conditions (step 8). To give the microfluidic device a flat surface, we put the model in the mold, poured PDMS, and squeezed out the excess PDMS (steps 10 to 11). The cross-section of the microvessel model is rectangular because the cross-section of the patterned photoresist is rectangular. Previously, we fabricated a vessel model with semicircular cross-sections using a reflow process with the patterned photoresist [8].

### 3.2. Femtosecond Laser and Mask Hybrid Exposure (FMEx)

Microvessels have been fabricated using semicircular photoresist patterns and light-cured resin, but the cross-sections of the fabricated channels were semicircular [8,21]. Therefore, these processes are not suitable for fabricating fine blood vessel models. So, to fabricate a capillary vessel and arteriole simulator with a submicrometer resolution and circular cross-section, we used a new 3D exposure method, specifically, two-photon absorption exposure by a femtosecond laser. However, since it takes a long time, this method is not suitable for exposing millimeter-scale areas. The typical microfluidic area used for the inlet or outlet port of the simulator is relatively large, that is, at a millimeter-scale. Therefore, these large areas were exposed using a mask aligner. The femtosecond laser exposure makes it possible to fabricate a part of the millimeter-scale structure with a submicrometer resolution in three dimensions.

#### 3.2.1. Femtosecond Laser Exposure System

We used a purpose-build femtosecond laser system [22]. This system uses a Ti:sapphire laser with a wavelength of 780 nm and pulse width of 140 fs (Chameleon XR-SK, Coherent, Inc., Glasgow, UK). The laser was focused using an objective lens with a numerical aperture of 1.40, magnification of 100×, and working distance of 130 µm (UPLSAPO 100XO, Olympus, Tokyo, Japan). Positioning of the sample was performed using a piezo stage (P-563 3 CD, Physik Instrumente Japan Co. Ltd., Tokyo, Japan). The positioning resolution was 4 nm, and the movable range was 300 µm in each direction (*x*, *y*, and *z*). A Karl Suss MJB3 mask aligner (MA-6, SUSS Micro Tec KK, Kanagawa, Japan) was used during fabrication. As in previous research [23], we used the negative photoresist KMPR (Nippon Kayaku Co., Ltd., Tokyo, Japan) and the positive photoresist PMER P-LA900PM (Tokyo Ohka Kogyo Co., Ltd., Kanagawa, Japan) as a negative control to fabricate capillary vessel simulators.

#### 3.2.2. Line Width Processing

**Figure 6:**
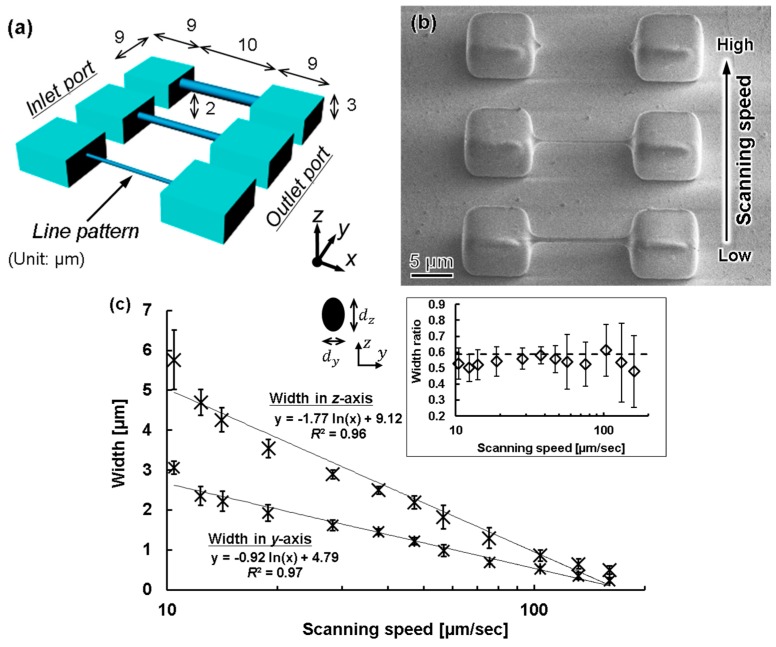
Measurement of scanning line width and thickness during femtosecond laser exposure. (**a**) Design concept for a microfluid device; (**b**) Top view SEM image fabricated 3D mold; (**c**) Relationship between scanning speed and line width and thickness by femtosecond laser exposure. Inset illustrates the ratio of each pair of line width dimensions, and the dashed line represents the theoretical ratio by the two-photon laser method, $\sqrt{\ln\left(2\right)/2}\approx 0.59$ [25].

As a preliminary experiment, we measured the minimum line width that could be processed when a PDMS block was exposed using the femtosecond laser, as shown in Figure 6. We varied the scanning exposure conditions to form various line patterns with the scanning laser, as shown in Figure 6a. The processed line pattern was observed using scanning electron microscopy (SEM; SPG-724, JEOL, Tokyo, Japan), and the width of the line pattern in the *x*-axis direction was measured. From the image (Figure 6b), obtained obliquely at 45°, the *z*-axis position estimates the width in the axial direction. The scanning line width was measured using a 100× objective lens, with the laser power set to 3 mW. The scanning speed was increased from 1 to 500 µm/s in the *y* direction. The obtained measurements are shown in Figure 6c. At 160 μm/s, the minimum possible line width was 250 nm in the *x*-axis direction and 490 nm in the *z*-axis direction. When the scanning speed was higher than 160 μm/s, the resist was not sufficiently exposed, and it was difficult to form the pattern, as shown by the top feature in Figure 6b. The line width decreased with an increasing scanning speed, and the mean ratio between widths in the *x* and *z* directions was 0.54 with a standard deviation of ±0.04. This approximates the theoretical number of the two-photon method, $\sqrt{\ln\left(2\right)/2}≒$ 0.59 [24,25]. Therefore, we could confirm that our fabrication technique with the femtosecond laser exposure had a reproducible resolution for a line width of 500 ± 100 nm and 240 ± 70 nm in the *z*- and *x*-axis directions, respectively.

#### 3.2.3. Experimental Procedure for FMEx

**Figure 7:**
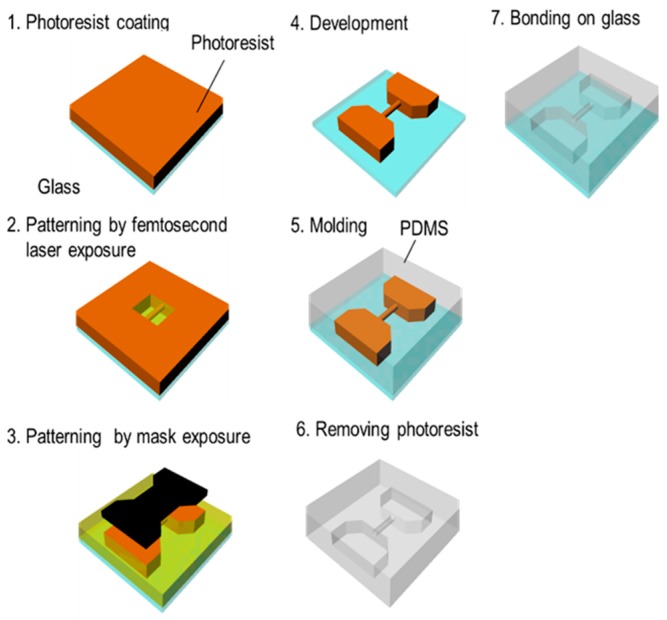
Microchannel model fabrication process using femtosecond laser and mask hybrid exposure (FMEx).

Figure 7 shows the process for fabricating a 3D capillary vessel simulator, which is summarized as follows (as before, step numbers correspond to those in the figure):

1. A 20-μm-thick film of KMPR or PMER was formed by spin-coating and pre-baking on a hot plate at 100 °C for 30 min.
2. A fine 3D microchannel was formed by femtosecond laser exposure.
3. The connection port, which introduces the liquid flow path, was exposed using the mask aligner.
4. The mold was heated using a hot plate at 65 °C for 1 min, 95 °C for 3 min, and 65 °C for 1 min. Then, the part was developed with PM thinner and rinsed with 2-propanol.
5. After replacing the mold with t-butyl alcohol, it was dried with a vacuum dryer and the pattern was transferred by pouring PDMS into the mold.
6. Remover PG was applied to remove the KMPR or PMER resist and form a hollow structure.
7. The finished microchannel was bonded to a glass substrate with plasma treatment.

## 4. Experimental Results

### 4.1. Photolithography Method

**Figure 8:**
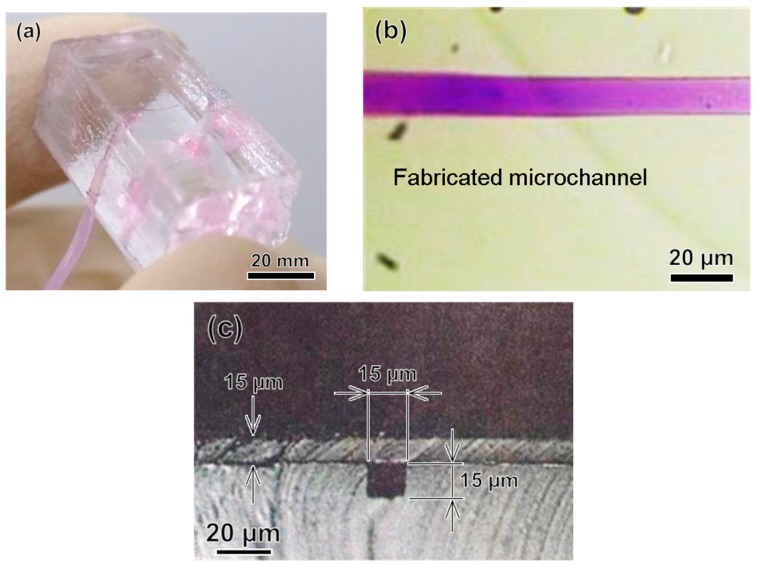
Images of the microchannel fabricated using classical photolithography. (**a**) PDMS base model with the microchannel; (**b**) Optical microscope image of the microchannel; (**c**) Cross-section of the microchannel.

Figure 8 shows a fabricated 3D microchannel in a PDMS block for modeling a microvessel. The channel was neither broken nor collapsed after the water transfer printing process, as shown in Figure 8b. The cross-section of the fabricated vessel model was square, as shown in Figure 8c, because the cross-section of the patterned photoresist was square. As noted above, the fabrication of a circular capillary vessel is quite complex using this exposure method [2,13], so the square cross-section was intentional.

**Figure 9:**
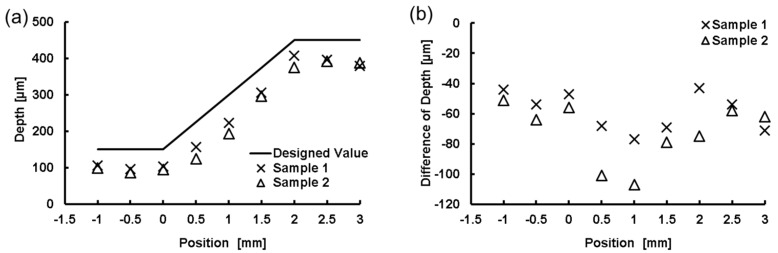
Measurement of microchannel depth. (**a**) Full thickness profiles of the designed and fabricated microchannels. Position zero is the inclination starting point; (**b**) Distributions of deviations of fabricated depth calculated by subtracting each fabricated value from the design value.

We created microchannels with various depths and measured the depth of the microchannel at different positions. The fabricated model was sectioned at different locations. Then, by using an optical microscope, we measured the depth of the microchannel at all sectioned locations, as shown in Figure 8c. Details of the designed and measured microchannel depths are given in Figure 9. In addition, we tested the flow of liquid in the microchannel by injecting a colored liquid into it via external tubes connected to the ports. The injected liquid flowed through the channel without leakage, as shown in Figure 8b.

### 4.2. FMEx Method

**Figure 10:**
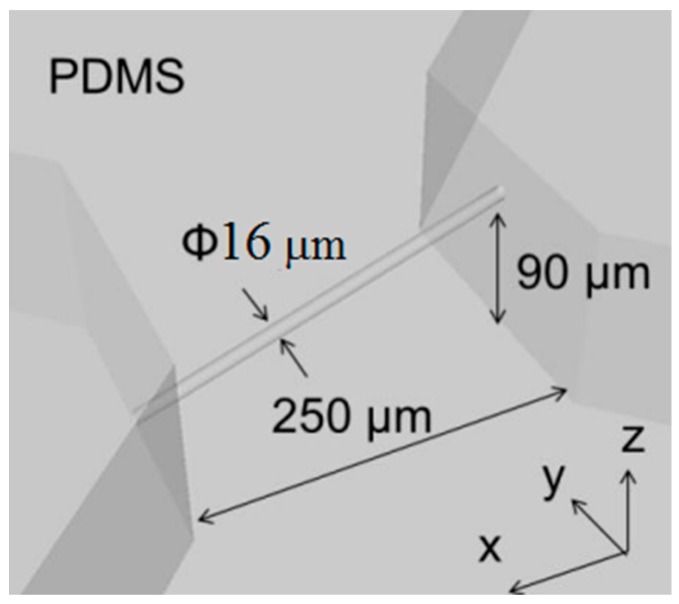
A CAD image design of 3D microfluidic devices for the replication of a microscopic vessel fabricated using FMEx.

**Figure 11:**
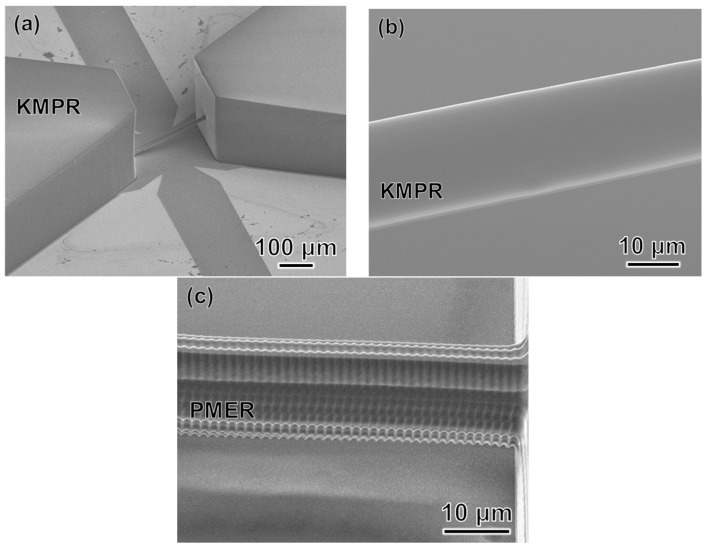
SEM images of the mold fabricated using FMEx: (**a**) Low magnification; (**b**) High magnification with a chemically amplified resist (KMPR); (**c**) High magnification with a standard resist (PMER).

**Figure 12:**
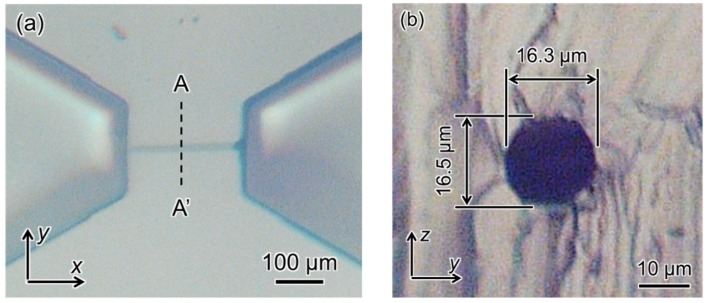
(**a**) Microscopic image of a PDMS microchannel mold fabricated using FMEx; (**b**) Cross-section of the microchannel at section A-A’.

Figure 10 shows a CAD image of our simple design for a 3D microfluidic channel. Processing conditions were a laser power of 3 mW, scanning speed of 50 μm/s, scanning interval in the *y*-axis direction of 0.99 μm, and scanning interval in the *z*-axis direction of 1.83 μm. SEM images of the produced mold are shown in Figure 11. Figure 11b is a 2700× enlargement of the mold prepared with the KMPR resist, which we compared with a mold prepared with a standard PMER positive resist (Figure 11c). The KMPR resist fabricated a microchannel with a smoother surface compared to the surface prepared with PMER. A cross-section of the microchannel formed with KMPR is shown in Figure 12. As defined by the length ratio between the *z*-axis and *y*-axis, the circularity of the channel using the FMEx method was 0.95.

## 5. Discussion

We developed two new methods for fabricating 3D microfluidic devices. First, we fabricated a microfluidic device using a conventional photolithography exposure method, which was limited to one plane, but with the assistance of PDMS molding and water transfer printing, we succeeded in forming a 3D capillary vessel with a rectangular cross-section at various depths in a slab-shaped model. With water transfer printing and 3D-printed models (steps 9 to 11 in Figure 5), we could easily control the depth of the microchannel to mimic the dimensions of a real blood vessel. The photolithographic exposure method was a suitable choice for the fabrication of a microfluidic device with a millimeter scale, as the time needed for exposure was short. However, to form a microchannel with a circular cross-section and smooth surface with this exposure method would be very complicated.

Second, we developed the FMEx method for fabricating 3D capillary vessel simulators with a diameter smaller than 20 μm. We employed the femtosecond laser exposure method to fabricate a millimeter-scale microchannel structure with a submicrometer resolution in 3D. By comparing the microchannels in Figure 11b, c, the latter of which was created by PMER, it can be seen that the FMEx method with the KMPR resist produced a much smoother surface. This smooth surface could be produced independent of minimum line width during femtosecond laser exposure scanning (Figure 11b). In addition, we compared our results with conventional studies on circularity. The model was sectioned and a cross-section image was taken with an optical microscope, as shown in Figure 12. In previous research, a capillary blood vessel model with a diameter of about 15 μm was produced by photolithography with overexposure, and it had a circularity of 0.84 [8]. The circularity produced by the FMEx method (Figure 12b) was improved, with a circularity of 0.95. Therefore, this study demonstrated that the FMEx method is a superior 3D modeling technique compared with photolithography using a standard photoresist.

In the fabrication of micrometer-scale channels (<20 µm in diameter) for mimicking blood capillaries, an important issue is how to connect an external tube to the microchannel for modeling capillary flow. In our research, we made our microchannel design with two regions to provide inlet and outlet ports for fluid, as shown in Figure 10, Figure 11 and Figure 12. Although the total device size included a micrometer-scale channel and two millimeter-scale connecting regions, we were able to easily make holes at these regions to connect external tubes and flow fluid through the microchannel. According to the effect of different exposure methods on the fabrication of a micrometer-scale channel, the mask exposure method was completed within a few minutes and it has the advantage of not being dependent on the exposure area.

The time of exposure was independent of model size and the time was short, up to a few minutes. On the other hand, it is hard to fabricate a large-scale model with the FMEx method, as both the laser exposure and removal of the photoresist after molding in PDMS take a long time. In this paper, we solved the problem of port connection by integrating masking techniques from classical photolithography. By using the FMEx method, we could create a 3D capillary vessel a few hundred micrometers in length and equip it with inlet and outlet ports.

**Figure 13:**
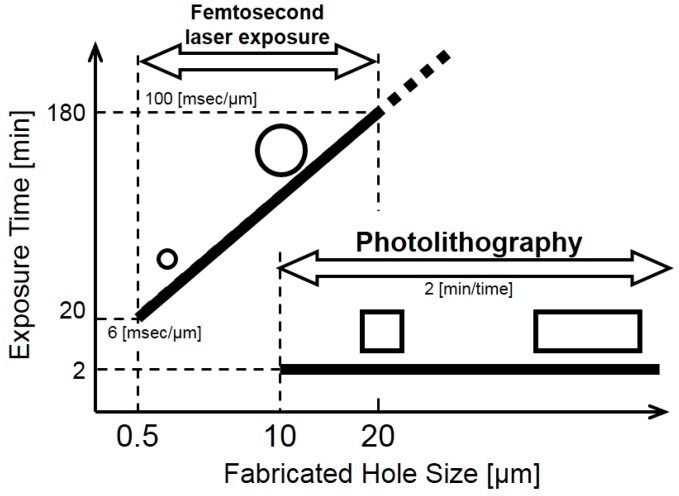
Schematic drawing of cross-sections of microchannels fabricated using femtosecond laser exposure and classical photolithography.

Let us consider the advantages and disadvantages of the photolithography and FMEx methods. Table 1 shows a comparison between the two methods used to fabricate 3D capillary vessels. Additionally, we present a schematic selection map of these fabrication methods in Figure 13. In cases where we need to fabricate a 3D capillary vessel with a small size, fine surface, and circular cross-section, the FMEx method is preferred. For simulating retinal microvessel surgery, such as microcannulation, with a model having large vessels >50 μm in diameter, a square versus circular cross-section makes no significant difference for surgeons [26]. Therefore, microvessels formed by the photolithography method would be sufficient during an evaluation of microcannulation. On the other hand, there is another need for the simulation of capillary vessels <20 µm in diameter in the superficial layer of the mucosa of the colon, which are especially targeted for cancer diagnosis by using a spectral endoscopy system [27]. In this case, both methods, photolithography and FMEx, can be used to form capillary vessel models with different depths from the surface. We used the photolithography method in the fabrication of capillary vessel models for an evaluation of the endoscopic imaging system [27].

Additionally, there is another need for the evaluation of deformability of RBC for the diagnosis of diseases. One approach for this evaluation is to measure the time of RBC passing through the simulated capillary vessel [28]. A capillary vessel simulator was used to flow in the simulated capillary vessel with a rectangular cross-section. By using the FMEx method, we can test and compare the 3D capillary vessel model with a circular cross-section and diameter similar to the geometry of an in vivo capillary vessel.

In the future, we will use the models fabricated by photolithography and FMEx methods, with the cooperation of expertise medical doctors, for quantitative observations of superficial capillary vessels. Additionally, we will use the model made by FMEx for the evaluation of RBC deformability. Thus, based on the application, we can choose the proper method from the two methods developed here for the fabrication of a 3D capillary vessel. Using our proposed methods, we can create multiscale transparent arteriole and capillary vessel models with circular cross-sections of a submicrometer to submillimeter diameter and lengths for evaluating the practice and rehearsal of surgeon skills and for developing new medical devices, such as spectrum endoscopy systems, and for studying hypotheses concerning the capillary vessel and its circulating cells.

## 6. Conclusions

In this paper, with the goal of creating micrometer-scale blood vessel models, we proposed 3D microfluidic devices fabricated using photolithography and FMEx. Photolithography with water transfer printing onto a 3D-printed model enabled us to quickly fabricate a 3D microscopic vessel with different depths and a large model size. The femtosecond laser exposure made it possible to fabricate part of the millimeter-scale structure with a submicrometer resolution in 3D. Also, we succeeded in obtaining a smooth surface independent of the minimum scanning line width of femtosecond laser exposure by using a chemically amplified resist that triggers acid diffusion. The acid diffusion phenomenon was essential for achieving a model with a smooth surface and circular cross-section. With the proposed FMEx method, we can create complex 3D capillary vessel models that realistically mimic blood vessels. Our proposed methods can make an important contribution to the medical field and create an alternative to the use of animals for surgical training and method evaluation.

## Figures and Tables

**Table 1:** Comparison between the femtosecond laser and photolithography fabrication methods.

Method	Femtosecond Laser Exposure	Photolithography
Dimension	3D	3D
Microchannel width	Max. 20 µm	Over 10 µm
Process time	Hours	Seconds
Cross-sectional shape of microchannel	Circular cross-section Circularity: 0.95	Rectangular
Total time of fabrication	Over 4 h	Less than 2 h
